# Bioprospecting Phenols as Inhibitors of Trichothecene-Producing *Fusarium*: Sustainable Approaches to the Management of Wheat Pathogens

**DOI:** 10.3390/toxins14020072

**Published:** 2022-01-20

**Authors:** Wiem Chtioui, Virgilio Balmas, Giovanna Delogu, Quirico Migheli, Safa Oufensou

**Affiliations:** 1 Dipartimento di Agraria, Università degli Studi di Sassari, Via E. De Nicola 9, 07100 Sassari, Italy; w.chtioui@studenti.uniss.it (W.C.); balmas@uniss.it (V.B.); qmigheli@uniss.it (Q.M.); 2 Istituto CNR di Chimica Biomolecolare, Traversa La Crucca 3, 07100 Sassari, Italy; giovanna.delogu@icb.cnr.it; 3 Nucleo di Ricerca sulla Desertificazione, Università degli Studi di Sassari, Via E. De Nicola 9, 07100 Sassari, Italy

**Keywords:** phenolics, *Fusarium*, wheat, Fusarium head blight, trichothecene mycotoxins, cereals, food safety, fungicides

## Abstract

*Fusarium* spp. are ubiquitous fungi able to cause Fusarium head blight and Fusarium foot and root rot on wheat. Among relevant pathogenic species, *Fusarium graminearum* and *Fusarium culmorum* cause significant yield and quality loss and result in contamination of the grain with mycotoxins, mainly type B trichothecenes, which are a major health concern for humans and animals. Phenolic compounds of natural origin are being increasingly explored as fungicides on those pathogens. This review summarizes recent research activities related to the antifungal and anti-mycotoxigenic activity of natural phenolic compounds against *Fusarium*, including studies into the mechanisms of action of major exogenous phenolic inhibitors, their structure-activity interaction, and the combined effect of these compounds with other natural products or with conventional fungicides in mycotoxin modulation. The role of high-throughput analysis tools to decipher key signaling molecules able to modulate the production of mycotoxins and the development of sustainable formulations enhancing potential inhibitors’ efficacy are also discussed.

## 1. Introduction

*Fusarium* spp. are found in various ecosystems, including agricultural soils, where they have a relevant impact on cereal crops [1,2,3,4,5]. Among the most important cereal diseases, fusarioses are incited by a complex of toxigenic species of the genus *Fusarium* [6]. Depending on the cereal type and the geographic area, the range of *Fusarium* species present may differ. *Fusarium culmorum* (W.G. Smith) Sacc., *Fusarium graminearum sensu stricto* (Schwabe), and *Fusarium pseudograminearum* O’Donnell and Aoki are considered as main pathogens of wheat [7,8,9,10,11,12]; whereas other species that are detected less frequently include *Fusarium acuminatum* Ellis and Everhart, *Fusarium avenaceum* Fr. (Sacc.), *Fusarium langsethiae* Torp and Nirenberg [13], *Fusarium poae* (Peck) Wollenw., and *Fusarium tricinctum* (Corda) Sacc. The presence of one or more *Fusarium* species also depends on other factors such as previous crops, management of cultural residues, environmental conditions, and cultivation techniques.

**Figure 1:**
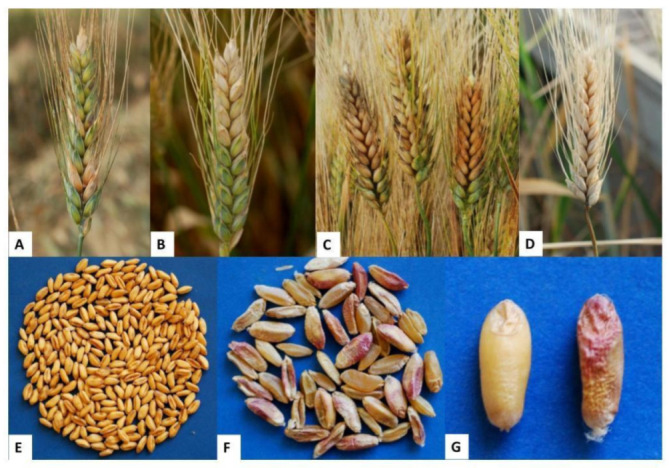
Symptoms of Fusarium head blight on durum wheat spikes (**A**–**D**) and kernels affected by fusariosis (**F**,**G**) compared to healthy kernels (**E**).

Fusarium head blight (FHB) is caused on wheat and other small grains, mainly by *F. graminearum* and *F. culmorum* [4,14] (Figure 1).

These fungi reduce yields and cause quality losses due to the production of mycotoxins [2,15]. *F. graminearum* and *F. culmorum* may produce zearalenone (ZEA) and type B trichothecenes; these include deoxynivalenol (DON) and its two acetylated forms: 3 acetyl-deoxynivalenol (3-ADON chemotype) and 15-acetyl-deoxynivalenol (15-ADON chemotype), as well as nivalenol (NIV chemotype) [16,17] and its acetylated form 4-acetylnivalenol or fusarenone-X (4-ANIV chemotype) [18]. DON is by far the most frequently detected mycotoxin in cereal grains worldwide [19,20,21,22], with incidences ranging from 50% in Asia to 76% in Africa [23].

Trichothecenes may occur in food and feed at high concentrations and have toxic effects on plants and animals [24,25,26]. They are phytotoxic in wheat, causing chlorosis, inhibition of root elongation, and dwarfism [27]. Moreover, livestock exposure to mycotoxins, including trichothecenes, may be responsible for direct production losses, such as milk production decrease in dairy cattle, but also indirect losses, as a consequence of reduced liver function immune responses, epithelial barrier function, and reproductive capacity [21,28]. Trichothecenes are also a cause of public health concern: these compounds elicit many adverse effects in humans, among which the most relevant are emesis, nausea, anorexia, abdominal pain, growth suppression, diarrhea, hemorrhage, and immunotoxicity [29,30].

*Fusarium* mycotoxins are among the most relevant causes of concern regarding chronic toxicity of natural food and feed contaminants and pose critical challenges in food toxicology [23,31]. Consequently, maximum contamination levels acceptable for DON in cereal-based food were set by the European Commission in June 2005 (EC no. 856/2005) and revised in July 2007 (EC no. 1126/2007) and by amending regulations [32,33,34,35,36]. These limits were fixed at 1250 μg/kg in unprocessed common wheat and 1750 μg/kg in unprocessed durum wheat for human consumption in the European Union (EC no. 1126/2006).

The efficient containment of *Fusarium*-associated disease and the reduction in food and feed trichothecene contamination poses a major challenge and requires integrated management approaches, spanning from the choice of tolerant cultivars, the adoption of crop rotation strategies, reduced nitrogen application, management of crop residues, and seed coating with biocontrol agents or antifungal compounds [23,37,38,39].

Fungicides bearing an azole unit are widely used in agriculture for the control of *Fusarium* species and their mycotoxins as they are generally inexpensive, have a broad spectrum of action and long stability [40]. Azoles inhibit the ergosterol biosynthesis pathway by blocking the sterol α-demethylase [41]. Despite their efficacy, though, if used incorrectly, they may induce a selective pressure on fungal populations, favoring the appearance of resistant mutants [42,43,44,45]. The frequent use of fungicides of the triazole family is also associated with a shift in the FHB-causing *Fusarium* species, e.g., by increasing the frequency of *F. avenaceum* (Fr.) Sacc. and *F. poae* while decreasing the population of *F. culmorum* and *F. graminearum* [46]. Studies on the *Fusarium* population showed the proliferation of highly aggressive strains and chemotypes, with high resistance to certain fungicides [46,47]. For example, a more aggressive and toxigenic *Fusarium asiaticum* O’Donnell, T. Aoki, Kistler and Geiser 3-ADON population has now replaced the previous NIV population in China. Similarly, in North America, a highly toxigenic population mainly formed by 3-ADON isolates of *F. graminearum* is replacing the existing 15-ADON population [47].

On the other hand, azole fungicides do not always warrant the decrease in mycotoxins in food and feed [48]. Under certain conditions, they may act as stress factors resulting in the induction of toxin biosynthesis [49,50,51,52,53]. Increased mycotoxin biosynthesis may take place when fungicides are distributed below the recommended dosage [54,55] or if they show differential fungicidal control of mixed FHB pathogen populations [56]. Moreover, chemical fungicides pose adverse effects on human health and on different components of the ecosystems, including water, soil, and non-target organisms [57,58].

Increasing efforts are now devoted to the design of alternative approaches to replace synthetic fungicides, particularly new classes of compounds capable of limiting the pathogenic and/or the mycotoxigenic potential of *Fusarium* spp., or able to enhance natural resistance mechanisms in the host plant [59,60]. For example, antioxidants have attracted considerable attention as they play a crucial role in the natural defense response of plants to oxidative stress caused by fungal invasion, and a strong, specific inhibitory activity was demonstrated for plant antioxidants (e.g., phenolic and polyphenolic compounds) against trichothecene-producing strains of *F. graminearum* and *F. culmorum* [61,62,63].

The objective of this review is to summarize the potentialities and limits of naturally occurring phenolic compounds as inhibitors of *Fusarium* spp. of agricultural interest, with emphasis on trichothecene producers affecting cereals: after a brief introduction on the structure and biosynthesis of trichothecene mycotoxins, the role of endogenous phenolic compounds in wheat defense reaction during fungal attack will be described. Then, the inhibitory effects of exogenous natural phenols on *Fusarium* vegetative growth and mycotoxin production will be illustrated. Finally, the possibility to design different combinations of phenolics and other natural compounds with improved activity against pathogenic *Fusaria* will be discussed.

## 2. Trichothecenes: Biosynthesis and Regulation

**Figure 2:**
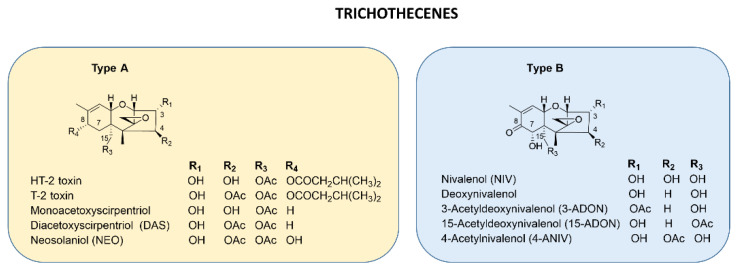
General structure of trichothecenes A and B.

**Scheme 1:**
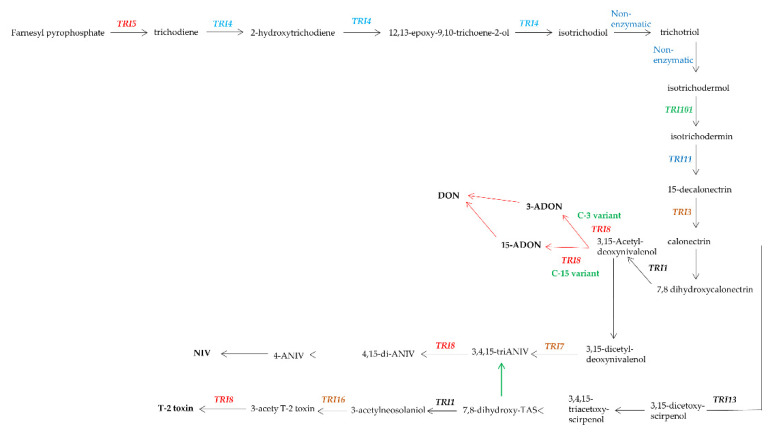
Trichothecene biosynthesis pathway.

Trichothecenes are esters of sesquiterpenoid alcohols positioned around a trichothecane tricyclic ring characterized by a double bond at C9–C10 and an epoxide at C12–C13 [64]. Trichothecene compounds are divided into four main groups, namely A, B, C, and D, based on their chemical properties and on the producing fungi. Trichothecenes synthesized by *Fusarium* spp. are included in groups A and B (Figure 2, Scheme 1).

Type A trichothecenes include: T-2 toxin and HT-2 toxin, diacetoxyscirpenol (DAS), scirpentriol (STO), 4-monoacetoxyscirpenol (MAS), and neosolaniol (NEO). These metabolites are mainly produced by *Fusarium sporotrichioides* Sherb., *Fusarium sambucinum* Fuckel, *F. poae*, *F. langsethiae*, and *Fusarium equiseti* Corda (Sacc.) [39,65,66]. Type B trichothecenes are characterized by a C-8 keto group and include: deoxynivalenol (DON), the acetyl derivatives (3-ADON), and 15-ADON), as well as nivalenol and fusarenone-X (4-ANIV) [18,67,68]. These compounds are predominantly produced in cereals by *F. culmorum*, *F. graminearum*, and *Fusarium crookwellense* L.W. Burgess, P.E. Nelson and Toussoun [17,69,70,71,72]. Types C and D share the presence of a carbonyl group attached to C-8 of the sesquiterpenoid backbone of trichothecenes. The presence of additional 7, 8 epoxides allows differentiation between type C from the other types. Type D contains a macrocyclic ring that connects C-4 and C-15 of the sesquiterpenoid backbone [67].

The precursor of trichothecene biosynthesis is the farnesyl pyrophosphate (FPP), an intermediate of the mevalonate pathway with a backbone of 15 carbon atoms [21,73]. The biosynthetic enzymes needed for trichothecene production are encoded by at least 15 *TRI* genes, which are located at three different loci on different chromosomes in *F. graminearum*: a 12-gene core *TRI* cluster, two genes at the *TRI1-TRI16* locus, and the single-gene *TRI101* locus [18,68,74]. Trichothecene production is driven by the expression of the *TRI5* gene, encoding the key biosynthesis enzyme trichodiene synthase, which cyclizes FPP to trichodiene (TDN), the first step in trichothecene biosynthesis [75,76]. TDN is then converted to calonectrin (CAL) following nine reactions that are sequentially catalyzed by TRI4 (a key multifunctional CYP58 family cytochrome P450 monooxygenase allowing four consecutive oxygenation steps in trichothecene biosynthesis, converting TDN to isotrichotriol), TRI101 (C-3 acetyltransferase), TRI11 (C-15 hydroxylase), and TRI3 (15-O-acetyltransferase). The reaction steps are found in *Fusarium* species producing type A trichothecenes (T-2 toxin and HT2) and type B trichothecenes (NIV and DON). In DON producers, CAL is hydroxylated at both the C-7 and C-8 positions by the cytochrome P450 monooxygenase TRI1 and deacetylated by the esterase TRI8 [77], leading to the formation of either 3-ADON or 15-ADON, followed by DON. A sequence variation in the coding region of the trichothecene biosynthetic gene *TRI8* was reported in *Fusarium* spp., indicating that differential activity of the TRI8 protein determines the 3-ADON and 15-ADON subchemotypes in *Fusarium* [78].

All these reaction steps catalyzing FPP to CAL are shared among *Fusarium* species that produce type A trichothecenes (T-2 toxin and HT2) and type B trichothecenes (NIV and DON). Two alternative pathways for NIV biosynthesis were observed for *F. graminearum*, involving either the TRI13-TRI7-TRI1-TRI8 pathway (and the CAL as a substrate) or the TRI13-TRI7-TRI8 pathway (with the 3, 15-ADON as the initial substrate) [79,80]. Depending on the type of trichothecenes produced, different chemotypes have been described: chemotype I, producing DON and/or its acetylated derivatives (3-ADON and 15-ADON), and chemotype II, producing nivalenol (NIV) and/or 4-acetyl-NIV [19].

Similar to other secondary metabolites, mycotoxins may be over-produced in response to external stresses, e.g., oxidative, nutritional, or light stress, as well as other environmental factors, such as pH, temperature, water activity, exposure to fungicides or plant secondary metabolites [81,82]. Temperature and water activity (a_w_) are the primary environmental factors influencing mycotoxin production by several *Fusarium* species [49,83,84,85]. For instance, *F. culmorum* and *F. graminearum* grow optimally at 15–25 °C in an a_w_ range of 0.98–0.99. Optimum DON production is situated at a_w_ = 0.97–0.99 for *F. culmorum* and at a_w_ = 0.98–0.99 for *F. graminearum*, all with an optimal temperature range of 15–25 °C [83].

Control of trichothecene production is driven by various transcriptional regulators involved in basal metabolic functions [86], e.g., the Pac transcription factor, which governs fungal responses to environmental changes such as pH [87,88,89] the velvet complex involved in response to light [90], and the *F. graminearum* FgAp1 factor, playing a role in response to oxidative stress [91]. Understanding the effect of each of these factors is essential to predict and prevent mycotoxin development.

During infection, plant cells respond to mycotoxin presence by a hypersensitive reaction that triggers the generation of reactive oxygen species (ROS), such as H_2_O_2_ and superoxide [24]. The oxidative properties of H_2_O_2_ modulate trichothecene biosynthesis [92,93] and induce increased expression of *TRI* genes [94,95]. However, *Fusarium* response to oxidative stress may vary depending on the ability to activate antioxidant defense responses and on the chemotype of the isolate: in vitro production of DON and 15-ADON by *F. culmorum* and *F. graminearum* chemotype I isolates can be enhanced upon H_2_O_2_ treatment, whereas NIV and 4-ANIV production by chemotype II isolates is reduced [96]. Similarly, differences in the detoxification ability were reported in the two chemotypes: isolates in chemotype I, when exposed to oxidative stress, react by increasing the catalase activity, resulting in a higher H_2_O_2_-degrading ability [96].

## 3. Role of Trichothecene Detoxification in Wheat Resistance against *Fusarium*

The ability of both *F. culmorum* and *F. graminearum* to spread in wheat is dependent on their potential to produce larger amounts of DON in culture [97,98] or in infected tissues [97,98,99,100], although this correlation is not always linear [97,101,102]. Trichothecenes also play an important role as virulence factors by inhibiting defense mechanisms activated by the plant [9]. Mutants of *F. graminearum* in which the ability to produce DON is impaired are able to infect but not to spread within the host plant [103,104,105,106].

Genetic improvement of wheat varieties by breeding or transgenesis to select wheat varieties resistant or partially resistant to *Fusarium* spp. is definitely the most sustainable approach to reduce the occurrence of these fungi and the contamination of grain with mycotoxins [82,107]. Plant resistance to FHB is a highly complex quantitative trait controlled by multiple genes [107,108,109,110]. The differing susceptibility of wheat cultivars to infection by *Fusarium* spp. is associated with different levels of mycotoxin contamination. This variability results from breeding programs, as well as agronomic and environmental cultivation conditions in individual countries [111]. Moreover, while the mechanisms by which abiotic stress may influence wheat resistance traits toward *Fusarium* spp. are still largely unknown, it is generally acknowledged that wheat would be more susceptible to *Fusarium* infection under future climate change conditions [112,113].

In wheat, two types of resistance to FHB were first described by Schroeder and Christensen [114]: type I (resistance to initial infection) and type II (resistance to fungal spread within the host tissue). Out of approximately 500 quantitative trait loci (QTLs) for FHB resistance mapped so far in wheat, most refer to type I and type II resistance, indicating their key role in controlling FHB. Some of these QTLs have been successfully applied in marker-assisted selection to improve FHB resistance [110,115].

Three additional types of resistance were defined: type III (ability to resist kernel infection); type IV (plant tolerance to infection and to the presence of DON and other secondary metabolites); and type V (resistance to the accumulation of mycotoxins in grain by converting them into non-toxic derivatives or by impeding the generation of toxic metabolites [39,116,117].

Given the key role of DON as a virulence factor for *Fusarium*, resistance to DON through detoxification or modulation mechanisms is considered as an innate component of FHB resistance. Kluger et al. [118] described the various metabolic routes involved in the detoxification of DON and reported a correlation between the efficiency of detoxification and a QTL for FHB resistance called *Fhb1*. Due to its pivotal role in wheat FHB resistance, *Fhb1* has been the subject of extensive map-based cloning studies to identify the causal gene. An early study has shown that *Fhb1* is involved in the conversion of DON into non-toxic DON-3-glucoside (D3G) [119]. Later, the *Fhb1* locus has been cloned from the resistant wheat cultivar Sumai 3 and shown to encode a chimeric lectin with two agglutinin domains relevant in carbohydrate binding. This protein also contains an ETX/MTX2 domain involved in pore forming, named PFT (pore-forming toxin-like) [120]. However, Yang et al. [121] found that TaPFT is also present in a number of highly FHB-susceptible wheat accessions, leading to reconsider the identity of *Fhb1*. Cloning of *Fhb1* has shown that its DON-detoxifying ability is not associated with PFT activity but rather with a putative uridine diphosphate (UDP)-glucosyltransferase that is also located on the chromosomal region introgressed from the cultivar Sumai 3 [120]. *Fhb1* was recently identified as an atypical disease resistance gene by two independent studies [122,123] reporting on the map-based cloning of *Fhb1*. In both papers, a critical deletion in the same gene coding for a reticulum histidine-rich calcium-binding-protein gene (His; also called HRC) was identified as the key determinant of *Fhb1*-mediated resistance to FHB in bread wheat. However, while these authors acknowledged the role of *Fhb1* in FHB resistance, they reached diverging conclusions on the causative allele: Su et al. [123] hypothesized that the *Fhb1*-mediated resistance is caused by a loss-of-function mutation; whereas Li et al. [122] concluded that this deletion results in a gain of function. Lagudah and Krattinger [124] explained the findings reached by these two apparently contradictory concurrent studies by conjecturing that the critical deletion may generate a dominant-negative effect. *Fhb2* is located on chromosome 6BS and confers enhanced type II FHB resistance [125,126]. Metabolomic and transcriptomic analyses of a recombinant inbred line carrying the *Fhb2*-resistant allele highlighted increases in defense-related compounds (phenylpropanoids, lignin, glycerophospholipids, flavonoids, fatty acids, and terpenoids), along with significant induction of genes encoding receptor kinases, transcription factors, signaling as well as mycotoxin detoxification proteins [127].

In the same QTL region, different putative defense-associated genes were identified, such as 4-coumarate: CoA ligase, callose synthase, basic helix loop helix transcription factor, glutathione S-transferase, ABC transporter-4, and cinnamyl alcohol dehydrogenase, suggesting that DON detoxification and cell wall reinforcement may be concurrently driven by *Fhb2*-regulated genes, thereby limiting the colonization of the wheat spike by the pathogen [127].

*Fhb5* is linked to a glutamate-gated ion channel, which is capable of triggering Ca^2+^ influx for early defense signaling in response to FHB [128,129].

The gene *Fhb7* from *Thinopyrum elongatum*, a wild relative of wheat used in breeding programs to improve cultivated wheat, encodes a glutathione S-transferase (GST). When introgressed into wheat backgrounds, *Fhb7* confers broad resistance to both FHB and *Fusarium* crown rot by detoxifying trichothecenes through de-epoxidation [130].

Manadalà et al. [131] demonstrated the efficacy of the barley *HvUGT13248* expressed in both bread wheat and durum wheat. The transgenic durum wheat displayed much greater DON-to-D3G conversion ability and a considerable decrease in total DON + D3G content in flour extracts, while the transgenic bread wheat exhibited a UGT dose-dependent efficacy of DON detoxification.

## 4. Major Plant Phenolic Compounds and Their Effect on *Fusarium*

**Figure 3:**
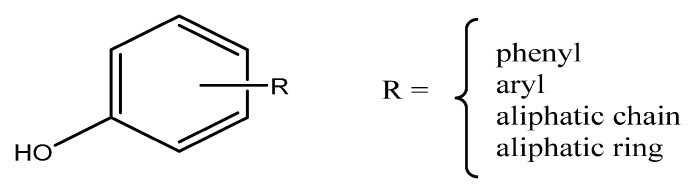
Schematic structure of the bioprospecting phenols.

Phenolic compounds contain at least one hydroxylated aromatic ring, with the hydroxyl group attached directly to the phenyl unit representing the core of the molecule. More oxygenated functionalities can be present and distributed in the other positions of the phenolic ring. Phenyl, aryl, aliphatic rings, and aliphatic chain, often containing hydroxylated functionalities, can be bound to the parent phenolic ring (Figure 3).

They contribute to various traits, such as pigmentation and resistance to pathogens in plants [132,133,134], and are generally present in food, spices, or food preservatives or belong to the list of generally recognized as safe (GRAS) compounds [135].

**Scheme 2:**
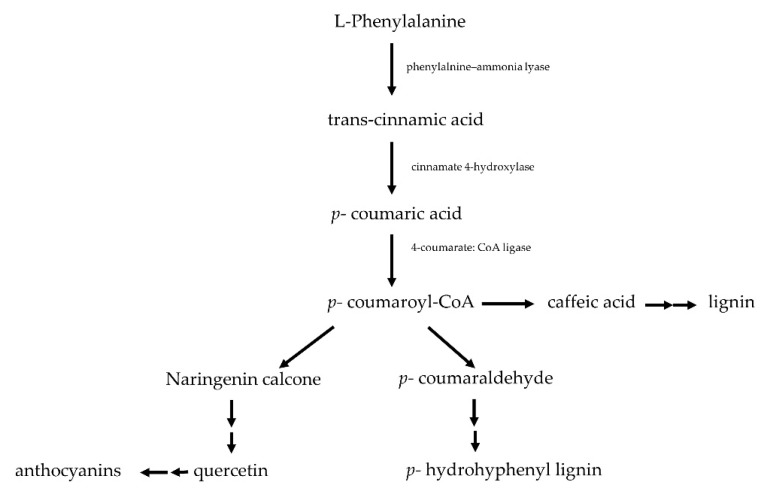
Phenylpropanoid pathway.

Phenolic compounds derive from the phenylpropanoid pathway [136], and their production is driven by phenylalanine ammonia-lyase (PAL), which converts phenylalanine into *trans*-cinnamic acid. This phenolic acid undergoes other enzymatic transformations, yielding a broad range of related phenylpropanoids [137] (Scheme 2).

**Figure 4:**
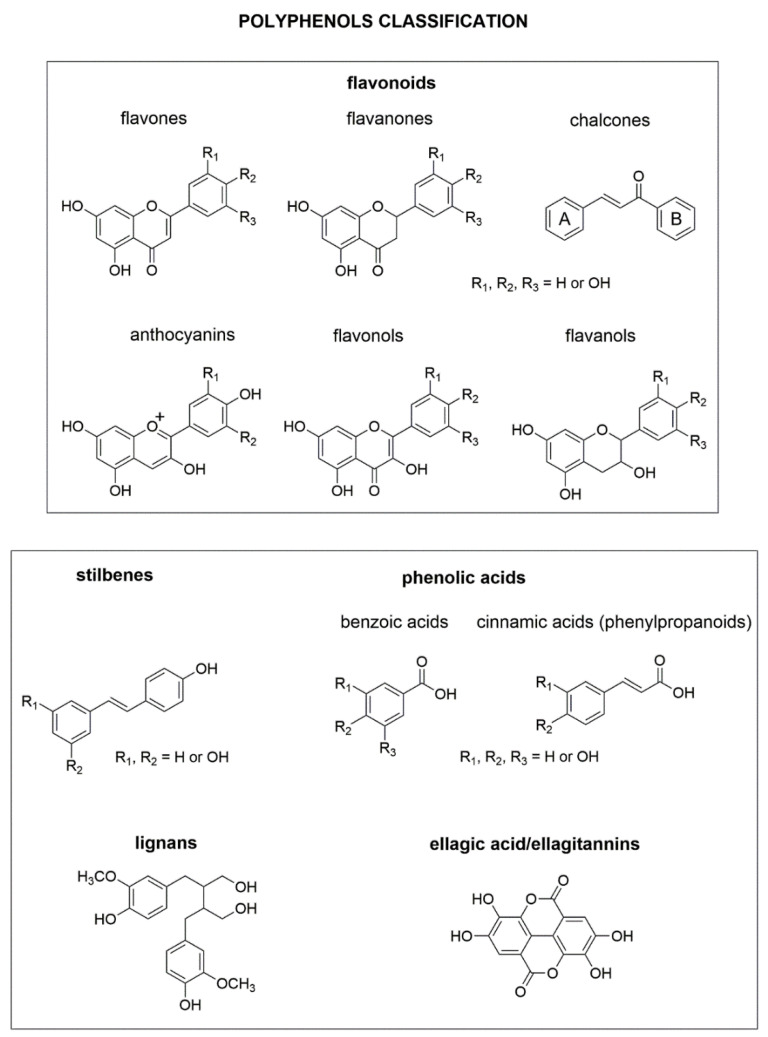
Classes of polyphenols.

They are chemically divided into two groups (Figure 4): flavonoid phenylpropanoids, including flavones, flavonols, flavanones, flavanols, anthocyanins, and chalcones; and non-flavonoid phenylpropanoids such as stilbenes, lignans, and phenolic acids [63] (Figure 4).

The majority of phenolic compounds are bound to the cell wall [138], which suggests their contribution to the preformed general defense system against potential pathogens [139,140]. The main role of flavonoids in plant defense mechanisms depends on their antioxidant properties [141,142,143,144], allowing them to quench ROS generated by both the pathogen and the plant during the infection process [145]. In addition, flavonoids, similarly to other non-flavonoid compounds such as cinnamic acids, are thought to take part in the reinforcement of plant cell walls and act as a physical barrier against fungal infection [146]: they protect plant cell wall integrity by hampering the activity of plant cell wall-degrading enzymes secreted by pathogens. Flavonoids are also known for their inhibitory activity toward fungal spore development, hyphal elongation, and fungal biofilm formation [109,147].

**Figure 5:**
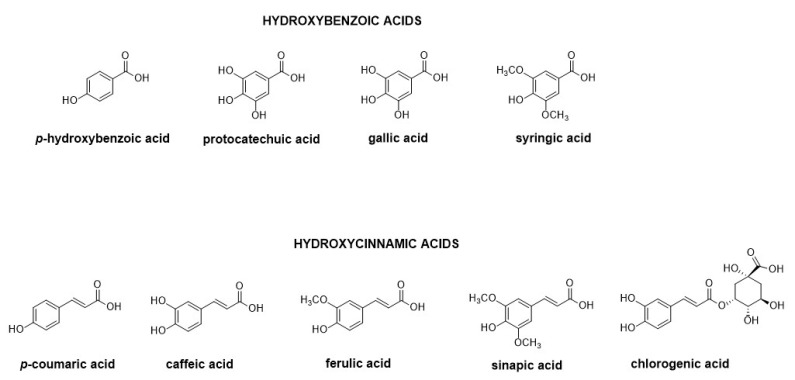
Structure of some representative phenolic acids with reported antifungal activity.

Phenolic acids form one of the main classes of non-flavonoid phenylpropanoids. Based on the number and position of hydroxyl groups on the aromatic ring, they can be divided into two main groups: the hydroxybenzoic acids and the hydroxycinnamic acids [148] (Figure 5).

Benzoic acid derivatives include gallic, *p*-hydroxybenzoic, syringic, protocatechuic, and vanillic acids, while caffeic, chlorogenic, *p*-coumaric, ferulic, and sinapic acids are included in the group of cinnamic acid derivatives (Figure 5). Cereals contain phenolic acids in both soluble (free) and insoluble (cell-wall-bound) forms [149]. Soluble phenolic acids include either free acids or esterified to sugar conjugates, whereas insoluble phenolic acids are conjugated to several polysaccharides and to lignin through ester and ether bonds. The soluble forms are compartmentalized within the vacuoles, while the insoluble forms are incorporated in cell walls [63,150,151,152].

Species, cultivar, and environmental conditions determine phenolic richness and composition in cereal grains through both constitutive and induced biosynthesis [153]. They likely reduce mycotoxin accumulation in plants, including trichothecenes [154] and fumonisins [155,156,157]. It is generally acknowledged that the fungus-plant interaction involves oxidative stress with the production of radical oxygen species (ROS) that enhance the biosynthesis of mycotoxins. The antioxidant metabolites present in cereal grains can play a crucial role in the resistance to *Fusarium* and in the production of mycotoxins [60,63,142]. Among phenolic acids, cinnamic acid derivatives accumulated in the kernel and well known as antioxidants are considered as the main contributors to FHB resistance [59,109,153,154]. Reactive oxygen species (ROS) are generated by fungi during their metabolic activity playing a crucial role when phytopathogenic fungi interact with plant cells. Gallic acid, a widespread plant metabolite, exhibits antioxidant activity interfering with ROS as a scavenging agent and produces cell apoptosis in the organism that generates ROS. In virtue of the metal-chelating properties of gallic acid due to the presence of hydroxyl groups in the aromatic ring, gallic acid might promote radical production exhibiting pro-oxidant activity. This behavior may appear in some conditions that depend on the concentration of the acid and in the presence of transition metals (i.e., Cu^2+^ and Fe^2+^). Pro-oxidant activity can accelerate damage to sensitive parts of the cell such as DNA, proteins, carbohydrates molecules, provoking the death of the organism [158].

## 5. Antifungal Activity of Exogenous Phenolic Compounds on *Fusarium* Vegetative Growth

Phenolic acids are common metabolites in plants and exert toxic effect on diverse fungi, including *Fusarium* species [60,61,62,63,109,132,143,150,151,152,159]. In cereal grains such as wheat, corn, rice, barley, sorghum, rye, oat, and millet, the predominant phenolic acids include ferulic acid, dimers of ferulic acid, *p*-hydroxybenzoic acid, sinapic acid, cinnamic, and vanillic acid [62,63,109,143,153,159]. A higher concentration of phenolic acids was observed in *Fusarium*-resistant wheat and corn plants than in susceptible ones, thus identifying these compounds as biomarkers of plant resistance [60,132,143]. The antifungal effect of phenolic acids was assayed in vitro by artificial amendment of each compound to the pathogenic fungi. According to the species of *Fusarium* on which the exogenous phenolic compounds are tested and on their concentration level, different antifungal activity was observed [153,160,161].

The bioactivity of phenolic compounds mainly depends on their ability to affect cellular membranes, with consequent impairment of cellular ionic homeostasis, acidification of vacuolar and cytosolic pH, and ultimately the destruction of structural cellular integrity [162,163,164,165,166].

Chlorogenic acid or 5-O-caffeoylquinic acid (CHLO), generated by the esterification of caffeic acid (CA) with quinic acid, is a cinnamic acid derivative (Figure 5). It is one of the most widespread soluble phenolic compounds in the plant kingdom and represents a key component of the plant defense mechanism against *Fusarium* [33,143,164,167]. CHLO was found to be the main phenolic acid that *F. graminearum* is likely to cope with when it infects the ear [168,169]. Gauthier et al. [33] tested CHLO and one of its hydrolyzed compounds in vitro on both *F. culmorum* and *F. graminearum* at concentrations close to the physiological amount previously quantified in kernels by Atanasova-Pénichon et al. [169]. Both chlorogenic and caffeic acids reduced fungal growth. CHLO showed a moderate antifungal effect with LC_50_ values > 10 mM, while caffeic acid was significatively more toxic. However, there is great variability in sensitivity to phenolic acids among *Fusarium* strains [33]. When comparing results obtained in the same conditions by Gauthier et al. [33] and Ponts et al. [59], it appears that *F. culmorum* strains (LC_50_ between 8.8 and 10 mM) are likely less susceptible to caffeic acid than *F. graminearum* (LC_50_ between 4 and 10.1 mM) [63]. Lately, Gauthier et al. [170] investigated caffeic acid (0.5 mM) on *F. avenaceum* at different pH conditions in liquid medium: caffeic acid inhibited only 10% of the growth at pH = 6 while at pH = 3, the fungal biomass was increased upon exposure.

Similarly, ferulic acid has a remarkable antifungal effect on *Fusarium* species. Boutigny and coworkers reported that ferulic acid reduces fungal biomass of *F. culmorum* by 39% at 2.5 mM and by 85% at 5 mM [154], whereas Pani et al. [161] found a significant inhibition of *F. culmorum* at the concentration of 0.5 mM. Ferulic acid is also reported to inhibit fungal growth in *F. graminearum*: 0.7 mM of ferulic acid reduced fungal growth by 50%, while 0.5 mM had no significant effect, albeit inhibitory concentrations are often strain dependent [59].

The fungistatic effects of phenolic acids on *F. graminearum* were ranked in ascending order of toxicity as follows: chlorogenic acid < *p*-hydroxybenzoic acid < caffeic acid < syringic acid < *p*-coumaric acid < ferulic acid: therefore, cinnamic-derived acids appear as more toxic compared to benzoic acid-derived ones [59,154,161].

Under certain conditions, fungal biomass can be increased by sublethal doses of ferulic, caffeic, or coumaric acid [170]. For example, ferulic acid applied at 0.5 mM induced an increase in fungal biomass of *F. langsethiae*, while at 1 mM, it reduced its growth [171]. In contrast, some phenolic acids display moderate effects on fungal growth: *p*-hydroxybenzoic acid has a minor effect at concentrations > 15 mM, reducing by 50% the growth of *F. graminearum* [59,153].

**Figure 6:**
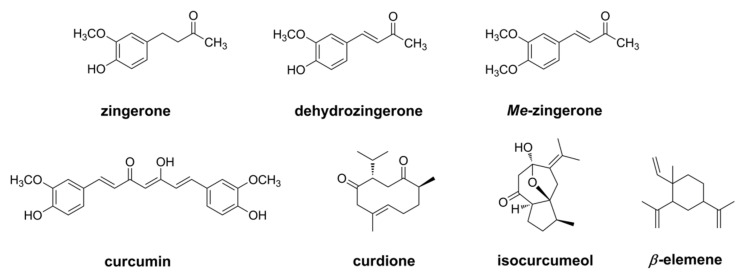
Structure of different compounds derived from *Zingiber officinale* and *Curcuma longa*.

Two phenylpropanoids, zingerone (4-(3-methoxy-4-hydroxyphenyl)-butan-2-one) and dehydrozingerone (Figure 6), are constituents of *Zingiber officinale*, structurally and biologically related to curcumin, with marked antifungal and antibacterial activity [172,173,174].

Pani et al. [161] studied comparatively the antifungal effect of dehydrozingerone and Me-zingerone on *F. culmorum*: both reduced fungal growth by >50% at 1.5 mM, but dehydrozingerone retained its inhibitory effect at 1 and 0.5 mM, whereas Me-zingerone had a stimulating effect on vegetative growth when applied at 0.5 mM. Tested at 0.5 mM, zingerone reduced by 83% the fungal growth and by 33% the DON production [161].

Chen et al. [175] tested different compounds derived from *Curcuma longa*, including curdione, isocurcumenol, curcumenol, curzerene, β-elemene, curcumin (Figure 6), germacrone, and curcumol at 0.5 mg/mL, by calculating the percent inhibition of mycelial growth in the untreated control. All these compounds displayed an inhibitory effect toward *F. graminearum*. Curdione showed an inhibitory rate of > 50%. The inhibitory effect of curdione in combination with isocurcumenol and β-elemene (tested at 0.25 mg/mL for each component) was 100%, while curdione combined with curcumin, curzerene, curcumenol, curcumol, and germacrone allowed inhibition rates of 93.6%, 88.9%, 82.7%, 63.6%, and 56.4%, respectively. Their toxicity involved fungal cell membrane disruption and inhibition of ergosterol biosynthesis, respiration, succinate dehydrogenase (SDH), and NADH oxidase activity [175].

Significant antimicrobial activity against pathogenic microorganisms has also been reported for thymol [5-methyl-(1-methylethyl) phenol], a natural monoterpene phenol found primarily in thyme, oregano, and tangerine peel [176]. Gao et al. [177] studied the hyphal growth, the conidial production, and germination of 59 isolates of *F. graminearum* under thymol treatment: the mean EC_50_ value for *F. graminearum* was 26.3 μg/mL. The molecular structure of thymol is responsible for its ability to dissolve and accumulate within the cell membrane, causing its destabilization, which has been related to the disruption of proton transfer efficiency [178]. In *F. graminearum*, the antifungal activity of thymol has been related to cell membrane damage as a consequence of lipid peroxidation and the disturbance of ergosterol biosynthesis [177]. Accordingly, thymol is reported to induce damages on the membrane integrity and the cell wall of other microorganisms such as *Candida* sp. [178], *Saccharomyces cerevisiae* [179], and *Bacillus cereus* [180].

Magnolol (1,5,5′-diallyl-2,2′-dihydroxybiphenyl), a natural hydroxylated biphenyl isolated from *Magnolia officinalis*, displays a wide range of biological activities [181]. Tested at 1.5 mM, magnolol exhibits a marked fungicidal activity in vitro toward *F. culmorum*; while a progressive decline in its activity has been observed at 1.0, 0.5, and 0.25 mM, the antifungal effect of magnolol remains significant at 0.25 mM [166,182]. Magnolol interacts with ergosterol in the cell membrane, inducing a partial disruption of its structure [183]: cell wall components, such as β-1,3-glucans, have been proposed as potential targets of magnolol, similarly to fungicides belonging to the echinocandin family [184]. Incidentally, magnolol is also potentially applicable to control human fusarioses: when tested on a collection of representative isolates of *Fusarium oxysporum* Schlechtend. emend. Synd. and Hans., *Fusarium solani* (Mart.) Sacc. and *Fusarium verticillioides* (Sacc.) Nirenberg of clinical and ecological concern, magnolol displayed a fungicidal activity similar to that shown by fluconazole (1–50 μg/mL), a fungicide widely used in treating fungal infections on humans [165]. Honokiol showed an even stronger antifungal activity than its isomer magnolol at 0.5 mM against *Fusarium* spp. [165]. The role of honokiol as an activator of mitochondrial ROS by dysfunction and depolarization of mitochondrial membrane potential in *C. albicans* has been highlighted [185]. Honokiol is also thought to burden the high content of pro-oxidant iron ions in yeast by sequestration [186]. Some differences between magnolol and honokiol in safety and toxicology have been reviewed by Sarrica et al. [187].

The efficacy of flavonoids as inhibitors of fungal growth has been referred to as their ability to react with nucleophilic amino acids in fungal proteins [188]. Compared to LC_50_ values described for phenolic acids, those detected for flavones and flavanones against *Fusarium* species, including *F. culmorum* and *F. graminearum*, are substantially weaker.

The promising ability of flavonoids to inhibit spore development and mycelium elongation of plant pathogens has been the subject of some studies [146,189]. Unsubstituted flavones and flavanones (with LC_50_ values comprised between <0.05 and 1.6 mM against *Fusarium* species, including *F. culmorum* and *F. graminearum*) display a higher antifungal activity than hydroxylated flavones (e.g., flavonol), with an LC_50_ in the 2.9–4.8 mM range [63]. Medical research has also focused on flavonoids as potential alternatives to synthetic drugs against human fungal pathogens displaying resistance to commonly used antifungal agents (e.g., triazoles).

Benzoxazinoids, a group of secondary metabolites present in several cereals, such as rye, wheat, and maize, play a key role as allelochemicals in the defense against predators and pathogen infection [190]. Their antifungal activity has been reported [181,182,183,184,185,186,187,188,189,190,191,192,193], and their role in wheat resistance to *Fusarium* spp. is being increasingly highlighted [134].

Inhibition of colony growth, of cell wall and membrane constituents (such as ergosterol and glucosamine), and alterations in enzyme activity with a consequent reduced biomolecular synthesis are all indicators of mechanisms involving the inhibition of cell multiplication. As previously mentioned, the inhibitory behavior of phenolic compounds depends on their ability to disrupt the integrity of the plasma membrane and to induce mitochondrial dysfunction, leading to metabolic stagnation [154,194]. For example, curcumin may disrupt the synthesis of critical proteins and enzymes, leading to inhibition of *F. graminearum* growth: this compound downregulates D-glyceraldehyde 3-phosphate: NAD^+^ oxidoreductase (GAPDH); moreover, it inhibits the biosynthesis of ergosterol and suppresses the activity of B-nicotinamide adenine dinucleotide (NADH) oxidase and succinate dehydrogenase (SDH), thereby interfering with the tricarboxylic acid cycle as well as inhibiting adenosine triphosphate (ATP) synthesis in the mitochondria [175]. Ferulic acid, instead, acts on the cell membrane, inducing significant changes in intracellular ATP concentrations, a decrease in the intracellular pH, cell membrane hyperpolarization, a reduction in cell membrane integrity, and ultimately evident morphological alterations. Gallic acid exhibits both antioxidants as well as pro-oxidant features, displaying a double-edged sword behavior, which turns it into an efficient apoptosis-inducing agent [158].

Quite regrettably, despite the powerful antimicrobial potential of these compounds, their poor delivery and bioavailability, coupled to the scarce stability, especially in the case of curcumin, do not allow them to reach the biological target at the bioactive concentration in plants.

## 6. Inhibition of Trichothecene Biosynthesis by Exogenous Phenolic Compounds

From a human health perspective, the main issue to consider in cereal protection is the capability of *Fusarium* to synthesize mycotoxins. Several phenolic compounds are able to modulate the production of mycotoxins in vitro in *Fusarium* species. However, their effect is highly variable depending on the class of mycotoxins, on the fungal species, the applied concentration as well as on the experimental conditions [153]. Some phenolics may even increase the biosynthesis of secondary metabolites in *Fusarium* spp.; therefore, it is essential to carefully consider each individual case: a partial inhibition of fungal growth is not necessarily correlated with the impairment of mycotoxin biosynthesis since the fungistatic activity could trigger secondary metabolic routes as a response to stress [195].

Cinnamic acid derivatives, such as ferulic acids, caffeic, *p*-coumaric, chlorogenic, and sinapic acid, are all efficient inhibitors of trichothecene mycotoxins produced by *F. graminearum* and *F. culmorum* [63,154].

Increased concentrations of ferulic acid reduce substantially most analyzed mycotoxins [153,196]. Bily et al. [150] reported a 57% inhibition of trichothecene production by *F. graminearum* in media supplemented with 0.25 mM ferulic acid. Moreover, antioxidant phenolic acids (e.g., ferulic acid) proved highly inhibitory toward both type A and type B trichothecenes [153,154], thereby suggesting a link with the evidence that accumulation of ferulic acid is positively correlated to *Fusarium* resistance in wheat varieties [153]. Ferulic acid inhibited the in vitro production of 3-ADON by 16–30% in *F. graminearum* and *F. culmorum* when applied at 0.5–1.0 mM [161,166]. This compound was also found to exert a transcriptional control, reducing the expression of key biosynthetic genes, namely *TRI5*, *TRI6*, and *TRI12* [82,154,197]. In the course of other studies, ferulic acid proved a powerful phenolic acid with anti-mycotoxigenic effects against various *Fusarium* species, including *F. graminearum*, *F. verticillioides*, *F. poae*, *F. langsethiae*, and *F. sporotrichioides* [63,153,171,198]. This compound and its dimeric forms play a key role in cereal resistance to *F. graminearum* and to DON accumulation and may also contribute to improving resistance to the infection by *F. avenaceum* and the associated contamination with enniatins [62,109,150,170]. The presence of dimeric forms of ferulic acid (DFAs) in the wheat kernel pericarp is associated with *F. graminearum* and *F. culmorum* resistance [62,150]. The main forms of DFAs are 8-5′-diferulic acid benzofuran, 8-0-4′-diferulic acid, 8-5′-diferulic acid and 5,5′-diferulic acid. DFAs are produced by coupling reaction of ferulate monomers catalyzed by peroxidase during cell wall deposition, conferring hardness to pericarp and resistance to fungal penetration. Fungal esterases and other hydrolytic enzymes attack the plant and induce the release of free DFAs from the plant cell wall polysaccharides. High concentrations of free DFAs during the plant-fungus interaction contribute to the inhibition of trichothecene biosynthesis by *Fusarium* [62].

Caffeic acid showed an inhibitory effect toward trichothecene: when tested at 1.0 mM, it led to complete inhibition of 3-ADON without affecting the mycelial growth of *F. culmorum* [161]. Similarly, 0.5 mM caffeic acid decreased the synthesis of type B trichothecenes by *F. graminearum*, whereas no significant effect on mycelium development was observed [86]. The ability of these compounds to impair mycotoxin production with no significant effects on fungal growth may be particularly useful for achieving mycotoxin control without applying selection pressure on resistant mutant populations [199]. Nonetheless, despite much evidence on the inhibitory effect of both ferulic and caffeic acid on trichothecene production by *Fusarium*, Ponts et al. [59] and Etzerodt et al. [200] highlighted a stimulating effect of these compounds on trichothecene biosynthesis. This could be explained by differences in strains, culture medium, and in vitro conditions of the experiment, reflecting fluctuating contexts in the delivery and bioavailability of the exogenous molecule.

Sinapic acid displays both antioxidant and antibacterial effects and plays an intriguing role as a preservative in foods [201,202]. Furthermore, it has been proposed as a resistance biomarker metabolite in cereals against *Fusaria* [203]. Kulik and coworkers [164] tested different levels of sinapic acid on both *F. culmorum* and *F. graminearum* under in vitro conditions, finding that exogenous application of this compound decreases the production of trichothecenes by both species, leading to 73.2–97.7% reduction at 3.6 mM. The expression of *TRI4*, *TRI5,* and *TRI10* genes was inhibited by sinapic acid, whereas an increase in ergosterol biosynthesis was observed. Thus, sinapic acid may bear the potential for its ability to limit mycotoxin contamination in food and feed [164].

Eugenol is another phenylpropanoid compound extracted from different plants with antifungal bioactivity toward *Fusarium* spp. [204,205,206]. Tested in vitro at 1.0 mM, eugenol induced complete inhibition of 3-ADON with no effects on vegetative growth in *F. culmorum* [161]. Similarly, the natural acetophenone apocynin (0.5 mM) reduced DON production of *F. graminearum* by 90% [166] and significantly reduced 3-ADON in *F. culmorum* without affecting fungal growth [161]. Both eugenol and apocynin proved efficient inhibitors of trichothecene also in field tests, albeit their bioactivity was transient and limited to the first post-inoculation stages [207].

**Figure 7:**
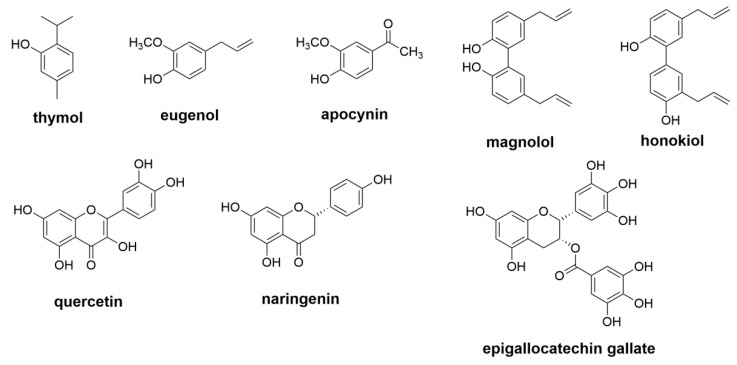
Structure of some phenols and flavonoids with inhibitory activity toward trichothecene biosynthesis.

Several studies illustrated the effect of flavonoids on mycotoxin production. Brown et al. [208] observed the ability of flavones to inhibit trichothecene production through the modulation of cytochrome P-450 monooxygenase-catalyzing conversion of TDN. Takahashi-Ando et al. [209] revealed that TRI4 is the potential target site of flavone and furanocoumarin in the inhibition of trichothecene biosynthesis. Bollina and Kushalappa [210] showed that naringenin and quercetin (Figure 7) induced complete inhibition of trichothecene biosynthesis in *F. graminearum* at early stages of incubation in artificial media. Bilska et al. [211] tested various amounts of exogenous flavonoids on different strains of *F. graminearum* and *F. culmorum*. Most flavonoids reduce trichothecene biosynthesis, but their effect depends on the fungal strain, the flavonoid compound, and its concentration. Quercetin was the most efficient compound, leading to a significant reduction (78.2% to 99.8%) in the accumulation of trichothecene, and the inhibition occurred at the transcriptional level. These data also confirm the role of the antioxidant activity on trichothecene inhibition: in virtue of differences in the structural feature and polarity existing between quercetin and naringenin, quercetin exerts a protective effect against bulk lipid oxidation, whereas naringenin fails.

The balance between lipophilicity and antioxidant activity can be a key factor in predicting the capacity of a phenolic compound to inhibit mycotoxin production. The ability of a compound to cross the fungal membrane lipids is mandatory to exert its antifungal/inhibitory activity. Fungal cultures are a peculiar system where both lipidic and emulsion systems coexist. In such a multicomponent environment, different physicochemical parameters, such as temperature, light, or pH, have a direct effect on lipophilicity and on the antioxidant capacity of phytochemicals. Therefore, correlating theoretical antioxidant potential and lipophilicity values with experimental data is far from being straightforward [161]. Nonetheless, the hypothesis that antioxidant properties of cereal metabolites can play a critical role in their anti-mycotoxigenic activity is consistent with the postulated activating effect of oxidative stress on the biosynthesis of mycotoxins [63]. Montibus et al. [212] emphasized the modulation of fungal secondary metabolism by oxidative stress and the enhancement of mycotoxin production, including DON, after exposure to reactive oxygen species. Thus, due to their ability to quench oxygen free radicals, antioxidant metabolites may reduce or suppress upstream signals such as oxidative stress that modulate toxin biosynthesis.

The toxicity of phenolic acids can also be linked to their interaction with various intra- and extracellular fungal enzymes, including phenol oxidases and several hydrolytic activities [63,213,214]. Moreover, Passone et al. [215] mentioned that antioxidant compounds interfere with mycotoxin production, probably indirectly via their capacity to perturb the membrane function and modify its permeability.

## 7. Effect of the Combination of Phenolic Compounds with Other Natural Products or Conventional Fungicides

Phenolic compounds isolated from natural sources present valuable antifungal properties, but their efficacy as inhibitors of mycotoxins and fungal growth is often strain and molecule dependent [33,154]. The scarce stability and/or solubility of the compound may also play a putative role. A possible strategy to improve their bioactivity is to combine natural compounds with other phenolic acids or benzo analogs or with conventional fungicides, resulting in the enhancement of antifungal activity against fungi [216].

In clinical practice, the synergetic use of antifungals is becoming popular to avoid resistance and reduce the required dosage of specific drugs [217]. Different studies described the efficacy of this method in containing *Candida* spp., a major group of fungal pathogens in humans [218,219,220,221]. By following the same approach, Dzhavakhiya and coworkers [216] found that the activity of azole and strobilurin fungicides can be significantly enhanced through their co-application with certain natural products against several economically important plant pathogenic fungi: thymol emerged as a potent chemosensitizing agent when combined with azoxystrobin on *Bipolaris sorokiniana* Shoemaker, *Phoma glomerata* (Corda) Wollenw. and Hochapfel, *Alternaria* sp. and *Parastagonospora nodorum* (Berk.) Quaedvl., Verkley and Crous at a non-fungitoxic concentration [216]. In addition, difenoconazole applied in combination with thymol significantly enhanced antifungal activity against *B. sorokiniana* and *P. nodorum*, while tebuconazole combined with 4-hydroxybenzaldehyde (4-HBA), 2,3-dihydroxybenzaldehyde inhibited the growth of *F. culmorum* at a significantly higher level than the fungicide alone [216].

Also, the combination of phenolic molecules and other natural compounds with differing modes of action may improve the inhibitory efficacy, as they could act in synergism with a multitarget effect [222]. For instance, Siranidou et al. [223] reported a synergistic antifungal effect of *p*-coumaric with ferulic acid in reducing the mycelial growth of *F. culmorum*. An equimolar combination of propyl gallate and thymol tested at a final concentration of 0.25 mM proved a strong inhibitor of trichothecenes both in vitro and in plants [224]. Oufensou et al. [166] tested an equimolar solution of thymol and magnolol, which had an additive effect on *F. graminearum*, possibly due to the different mode of action of the two compounds, or/and to the ability of one compound of the mixture to cross the fungal membrane, thereby improving the delivery of the other compound. Accordingly, plant extracts including various phenolic compounds and terpenes were highlighted as promising antifungal agents, the efficacy of which was attributed to a potential synergistic effect of the different components [225,226]. Recently, Montibus et al. [226] investigated the effect of maritime pine sawdust, a by-product from the industry of wood transformation, which includes, among other bioactive molecules, 11 compounds belonging to three families of phenolics, namely phenolic acids, lignans, and flavonoids, on various strains of *F. graminearum*. Pine sawdust tested at 500 mg/L proved extremely efficient, leading to a total inhibition of trichothecene production, with no fungal biomass reduction, for five out of six strains of *F. graminearum* tested.

Several compounds have different behavior in vitro and in plants. In vitro, the fungus is closely in contact with the potential inhibitor, whereas in plants, the effect of the compound is weaker due to the need to reach fungal cells within the colonized plant tissues. Lipophilicity and antioxidant activity of the inhibitor and composition of the carrier solution are key elements to magnify the effect of the potential inhibitor in plants. Phenolic compounds may be combined with essential oils to improve their bioavailability. The hydrophobicity of essential oils enables a better partition of phenolic compounds within the lipids of the cell membrane and mitochondria, thereby increasing their permeability and ultimately leading to the release of intracellular constituents [227,228] and to interference with many biological processes [229]. Wang et al. [230] showed that *Colletotrichum gloeosporioides* (Penz.) Penz. and Sacc. exposed to clove oil exhibits morphological and ultrastructural alterations, confirming the disruption of the fungal cell wall and of the endomembrane system, increased permeability, and loss of intracellular constituents. Therefore, investigations on the essential oils as co-formulants open a new scenario in the antifungal strategy, even though the reproducibility and stability of the essential oil mixture represent two elements of weakness in this approach. Nevertheless, essential oils are gaining popularity as safe and effective antifungal agents, in combination with other naturally occurring phenol exhibiting a different mode of action. For instance, Ochoa-Velasco et al. [231] reported antifungal effects of carvacrol and thymol below their MIC values against *F. verticillioides* and *Rhizopus stolonifer* (Ehrenb.) Vuill.

The ability to increase aqueous solubility is definitely a valuable aid to resolve the solubility problems of hydrophobic compounds, especially if the bioactive compound should be applied on the canopy by spray method. In this case, a right compromise between lipophilicity of the compound and wettability and complexation ability of the delivery composition is of paramount importance for the efficiency of the inhibitor/fungicide.

## 8. Sustainable Formulations for Bioprospecting Phenolic Compounds

Formulation technology plays a crucial role in the efficacy of the potential phenolic inhibitors: without a proper formulation, even the most effective compound is worth nothing [232]. Nowadays, formulation technology offers a wide choice of molecules, polymers, and materials; nevertheless, the search for sustainable agrochemical formulations is still open and strict rules concerning the safety of humans, animals, and the environment must be taken into account [233,234]. Many bioprospecting phenolic compounds are not water soluble or water dispersible [171], and this is a major drawback since the most common mode of delivery of any active ingredient into a crop is via spray applications of an aqueous solution or treatment of seeds with aqueous emulsions. Although many efforts have been devoted to the search for phenolic compounds effective against *Fusarium* spp., the medium used to solubilize the compound often lacks in safety for eventual application in the field. Dimethyl sulfoxide, acetone, and ethanol are the most common solvents used in vitro to assess the effectiveness of phenols. Usually, the concentration of the compound in these solvents ranges between 0.05 and 0.5 mM, which represent the minimum concentrations that allow the solubilization of the compound by preventing toxic effects due to the solvent present in the aqueous solution (1–10%) [153].

Tween 20, a non-ionic surfactant, improved dispersion of a hydrophobic curcumin derivative in aqueous solution, thereby allowing the aspersion of the aqueous fungicidal solution onto phytopathogenic fungi [235]. Tween 20 is a polysorbate containing lauric acid and 20 repeat units of polyethylene glycol distributed across four different chains. A nano and micro-emulsion of thymol and Tween 20 was used in combination with sunflower oil favoring the dispersion of the phenol in wheat plants [236]: complete FHB inhibition was achieved at 0.5% thymol, but phytotoxic effects were observed [236]. Among commercial polysorbates, Tween 20 is allowed in feed at a maximum concentration of 5000 mg/kg without any safety concern, while Tween 80 is generally used in vitro assay at the concentration of 10% (*v*/*v*) [237].

Other biomatrices recovered from waste of industrial activity or produced on a large scale have been assayed as safe formulating agents: collagen, chitosan, starch, cyclodextrins (CDs), carboxymethylcellulose (CMC), polylactic acid, polyethylene glycol (PEG) [232]. Water-soluble microcapsules made of a blend of collagen hydrolysates, CMC, and thyme oil were applied as a film on wheat seeds surfaces [238]: the authors investigated only the preparation of the microcapsule and their characterization in terms of water content, shelf-life stability, and release of the active ingredients.

CDs, cyclooligosaccharides obtained as by-products of starch degradation, are now produced by effective biotechnological processes in α-CD (six-cycloamylose units), β-CD (seven-cycloamylose units), and γ-CD (eight-cycloamylose units) [232] separately. Structurally, CDs are constituted by an amphiphilic torus with a hydrophobic interior cavity able to host lipophilic molecules. Due to a truncated conical shape and an external hydrophilic surface, CDs can form water-soluble inclusion complexes with lipophilic molecules or activate strong interactions with them, facilitating the delivery/solubilization of the molecule in aqueous solutions. Due to these properties and their non-toxicity, CDs have been largely used in medicine, food, and materials, and promising carriers in antifungal formulations were proposed [239]. The synergistic effect of CDs with phenol-based essential oils was observed against fungal pathogens [240,241]. Although phenolic molecules activate strong interactions with CDs, only few examples appeared in literature as emulsifier for in vitro antifungal assay [161,182] and as formulating agents in agriculture against *F. graminearum* and *F. culmorum* [207,242]. All the available examples are based on the use of 3 mM aqueous solution of β-CD as an emulsifier agent of phenols.

Efforts were devoted to the preparation of technologically advanced biomaterials where the fungicide is embedded in nanoparticles or hydrogels. These formulations improve the shelf-life of the fungicide, its delivery, and permeability through the fungal membrane, maintaining safety and health criteria. Among the naturally occurring biomatrices, nanosized chitosan particles and hydrogel chitosan-based are now considered suitable formulating agents [232].

Loron et al. [243] tested the starch octenylsuccinate (OSA-starch) and the chitosan as a matrix for the encapsulation of the curcumin derivative tetrahydrocurcumin (THC), demonstrating the antifungal and anti-mycotoxigenic properties of the encapsulated particles against *F. graminearum*. Both starch and chitosan spray-dried particles seemed to better protect THC and to extend its time of release, even though THC-loading aspects should be taken into account.

An ideal formulation should be inexpensive, environmentally sustainable, easy to distribute, and should present a shelf-life long enough for proper storage. Coating-forming agents may contribute to enhancing the properties of formulations. Although many promising bioformulations appeared in literature, unfortunately, no biomatrices have yet reached an advanced stage of development and commercialization to be applied in agriculture.

## 9. Structure-Activity Interactions

As previously mentioned, some phenolic compounds are reported as strong inhibitors of mycotoxin production without any effect on the fungal biomass [244]. Identifying molecules with specific molecular targets in the trichothecene biosynthesis pathway with no fungitoxic effects would be highly desirable to reduce the selective pressure on fungal populations, hence limiting the onset of resistant mutants. Given the fact that DON acts as a virulence factor, its inhibition may reduce the infection process and the development of the disease symptoms.

An early molecular docking study was carried out with the trichothecene 3-O-acetyltransferase TRI101 as the target protein. The ligand, a phenyl derivative of pyranocoumarin (PDP) extracted from *Psoralea corylifolia* seeds, showed a strong affinity toward the TRI101 by inhibiting the acetylation mechanism of the trichothecene and leading to the destruction of the “self-defense mechanism” of *Fusarium* sp. [245].

Pani et al. [182] investigated the mechanism of trichothecene inhibition by focusing on the binding mode of diverse naturally occurring phenols. Docking analyses were performed onto a 3D atomic-level protein model of the *F. culmorum* trichodiene synthase TRI5, based on the crystal structure of *F. sporotrichioides* TRI5 [246]. Docking analyses identified two sites (named site 1 and site 2) located on the surface of TRI5 *F. culmorum* as privileged sites for phenol-based hydrophobic ligands inhibiting trichothecene biosynthesis in vitro. Phenols with a long aliphatic chain and in dimeric form (i.e., hydroxylated biphenyls) interact simultaneously with sites 1 and 2. Propyl gallate, ellagic acid, magnolol, eugenol, and the eugenol dimer bind preferentially to sites 1 and 2 and far from the catalytic domain. With few exceptions, no-charged phenols interact with the same set of amino acids identified as: Gln68, Thr69, Tyr76, Trp298, Leu300, Cys301, Asp302, Ala303, His308, Phe329, Ala333, Gly336, Ala337, Val338, Trp343.

Aiming to provide further insight into the understanding of structure-activity relationship, Maeda et al. [244] have identified NPD352 [testosterone 3-(O-carboxymethyl)oxime amide-bonded to phenylalanine methyl ester], a TDN inhibitor identified from a chemical library of the RIKEN Natural Product Depository by chemical array screening using a recombinant trichodiene synthase tagged with hexahistidine (rTRI5) as a target protein. Unfortunately, the high lipophilicity of NPD352, its high molecular weight, and the high cost of production do not permit the development of the compound for a straightforward application in agriculture. The author also highlighted that, by optimizing the steroid skeleton, so to minimize endocrine perturbation and by modifying the side chains of the aromatic amino acids for higher activity, effective natural-like inhibitors of trichothecene biosynthesis may be developed in the future [244].

Another computational study on TRI5 protein has been recently carried out by Oufensou et al. [224]. A set of 15 naturally occurring compounds belonging to cinnamic acids, gallic esters, terpenes, phenylpropanoids, and 1 phenylethanone was selected for docking onto TRI5. Based on this protein model, the binding capacity of the selected compounds and of NPD352 [244] with the TRI5-inorganic pyrophosphate model (TRI5-PPi) was studied by comparing the most populated sites with those evaluated when the same compounds were docked with TRI5 containing the substrate (i.e., farnesyl pyrophosphate (FPP)). The five sites previously identified by Pani et al. [182] in the TRI-PPi model were also confirmed for the tested phenolic compounds, thereby confirming sites 1 and 2 as the privileged ones. Notably, NPD352 interacted with the same sites and with the same set of amino acids, providing further proof of the reliability of the in silico TRI5 model.

Recently, several computational analyses have been introduced for the prediction of drug targets in *F. graminearum* [247,248]. However, not enough effort for discovering novel natural drugs has been reported because of the unavailability of the crystal structure of drug targets.

## 10. Conclusions and Future Trends

*Fusarium* mycotoxins are an important challenge in agriculture worldwide, particularly in the cereal and grain production sector. Control of *Fusarium* spp. is largely based on the use of fungicides bearing an azole unit that is used for both plant and human therapy, as they are generally inexpensive, share a broad spectrum of action and long stability. In recent years, large-scale abuse of azoles in agricultural settings has been blamed as a major driver of the increasing resistance phenomena that are also being reported in human pathogenic fungi [40,249]. These concerns will certainly lead to drastic restrictions in the use and availability of active ingredients to cope with phytopathogenic fungi. Under such circumstances, natural phenolic compounds are becoming increasingly attractive, as they prove potent antifungal agents, when applied singly or in combination, with less or no toxic effect and differing mechanisms of action. Phenolic compounds with low/moderate molecular weight are rapidly metabolized by the natural microflora, thereby providing an essential alternative to industrial agrochemicals, which are often detrimental to the environment. Most of these compounds are widely used as food additives, as they are commercially available at a reasonable price [250].

The antifungal and anti-mycotoxigenic activity of different cinnamic acids, acetophenones, benzaldehydes, benzoic acids, phenylpropanoids, or hydroxylated biphenyls, has been widely reported over the last decades. Yet, their efficacy appears dose and strain dependent, often leading to inconsistent results, especially when moving from the lab to the field. Additional studies are required to highlight their in vivo activity, toxicity, and bioavailability through the design of sustainable formulations. This shall pave the way for the selection and identification of new fungal targets and possibly of new “anti-mycotoxin” molecules with no fungicidal effect, aiming to reduce the selection of resistant mutants [251].

The recent improvements in analytical platforms using integrated high-throughput technology, such as transcriptomics, proteomics, microbiomics, and metabolomics, providing multi-level omics data, may help to further identify relevant factors governing mycotoxin production and shall improve significantly our understanding of the mode of action of natural bioactive molecules to be used as new eco-friendly targets to mitigate the issue of food and feed contamination. A combination of ^1^H NMR and LC-QTOF-MS analyses tools have been implemented by Atanasova-Pénichon et al. [86] to explore the interdependence between the biosynthetic pathway of DON and the central metabolism, comparing the exo- and endo-metabolomes of *F. graminearum* grown in different culture media amended with phenolic compounds as toxin-inducing or -repressing conditions. Metabolome alterations induced by DON-producing *Fusarium* have also been evidenced aiming to the characterization of key plant metabolites that may contribute to resistance to fusarioses or interfere with DON accumulation [109,252,253,254].

Despite our growing understanding, it is evident that further research will continue to more accurately define the food safety risks management associated with new eco-sustainable molecules with different mechanisms of action and to shed light on the factors contributing to the success of these versatile and interesting compounds as plant protectants. Given the importance of phenolics in industry, food safety, and human health, and the growing interest in understanding the regulation and expression of the fungal secondary metabolome, this field is likely to represent a fertile prairie for the next generation of researchers.

## Data Availability

No new data were created or analyzed in this study. Data sharing is not applicable to this article.
